# Letter on Update to the Vitamin C, Thiamine, and Steroids in Sepsis (VICTAS) Protocol

**DOI:** 10.1186/s13063-020-04289-z

**Published:** 2020-04-22

**Authors:** Mark A. Frommelt, Pierre Kory, Micah T. Long

**Affiliations:** 1 Department of Medicine, University of Wisconsin, 1685 Highland Avenue 5158 Medical Foundation Centennial Building, Madison, 53705-2281 WI USA; 2 Department of Anesthesiology, University of Wisconsin School of Medicine and Public Health, B6/319 UW CSC, 600 Highland Ave., Madison, WI 53792-3272 USA

To the Editors:

Vitamin C, thiamine and steroids in sepsis (VICTAS) is an increasingly used therapy at centers around the world according to emerging clinical data. A multicenter randomized trial is crucial to more accurately determine the safety and efficacy of this therapy, and we eagerly await results from the VICTAS study [1]. Therapy with vitamin C, thiamine and steroids may decrease oxidative stress and inflammation in sepsis while improving mitochondrial function and stabilizing the endothelium. There may be other benefits, such as maintained catecholamine synthesis, improved aerobic metabolism, and decreased septic immune suppression [2, 3].

Considerable evidence supports the widespread belief that earlier interventions in sepsis contribute to improved clinical outcomes [4]. Unlike observational trials, randomized controlled trials in sepsis are faced with accounting for this urgency while providing time to stabilize the patient, update family, obtain and document informed consent, randomly assign patients, and administer the intervention (or placebo). Just as we would never defer starting antibiotics until morning rounds, delays in correcting deranged metabolic physiology with VICTAS may limit benefit. Furthermore, antioxidant therapy may offer the most benefit during the initial and most injurious oxidative burst. Later therapy may impair oxidative signaling necessary for cellular adaptation to stress [5].

We recently performed a retrospective cohort study of 208 patients in septic shock at our institution, all meeting multiple strict inclusion criteria; the 79 patients who received VICTAS must have done so within 24 h of intensive care unit (ICU) admission. We found that the ICU mortality ratio—the ratio of the observed to the APACHE (Acute Physiology and Chronic Health Evaluation)-predicted ICU mortality—of patients who received VICTAS linearly increased with delays in treatment from initial sepsis presentation (Fig. 1). Furthermore, in patients who received vitamin C, thiamine and steroids in sepsis within the Centers for Medicare & Medicaid Services Studies 6-h sepsis bundle time line, we found that the APACHE-adjusted ICU mortality was significantly reduced when compared with that of patients who received standard care (odds ratio [OR] 0.075 [0.0, 0.59], *P* <0.01). In contrast, for patients beginning VICTAS more than 6 h after sepsis presentation, we did not find a statistically significant difference in APACHE-adjusted ICU mortality (OR 0.66 [0.27, 1.50], *P* = 0.33).

**Fig. 1:**
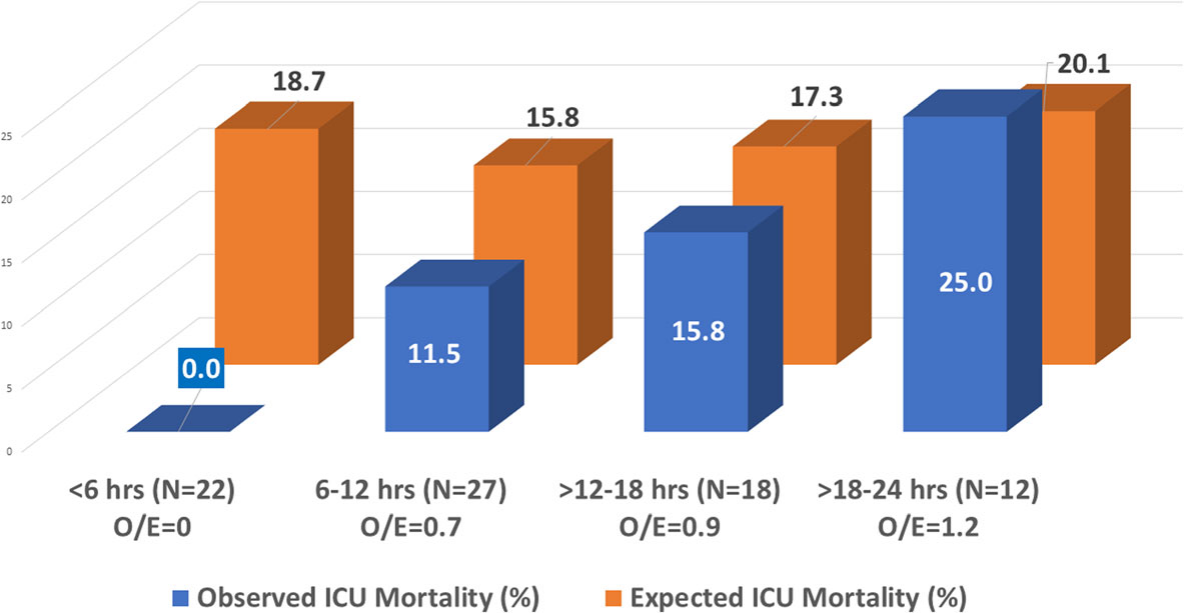
Intensive care unit (ICU) mortality and delay in vitamin C, thiamine and steroids (VICTAS) administration. “Hrs” refers to time from sepsis presentation to VICTAS initiation. “O/E” refers to the ratio of observed ICU mortality to the expected ICU mortality derived from APACHE (Acute Physiology and Chronic Health Evaluation) score

Patients can be enrolled in the VICTAS trial up to 24 hrs after evidence of sepsis-related organ dysfunction as evidenced by vasopressor requirement or pre-defined respiratory support needs. After organ dysfunction is identified, a further 4 hrs is allotted before patients must be given VICTAS or placebo therapy [1]. This suggests that a significant number of patients enrolled in the trial may receive VICTAS after extensive delays from presentation of sepsis, which may prove to be a limitation in assessing its efficacy. Therefore, we strongly suggest a planned subgroup analysis evaluating the relationship between timing of therapy and clinical outcomes.

## Data Availability

The datasets used or analyzed (or both) during the current study are available from the corresponding author on reasonable request.

## Abbreviations

APACHE: Acute Physiology and Chronic Health Evaluation
ICU: Intensive care unit
OR: Odds ratio
VICTAS: Vitamin C, thiamine and steroids

## References

[1] Lindsell CJ, Mcglothlin A, Nwosu S (2019). Update to the Vitamin C, Thiamine and Steroids in SsSepsis (VICTAS) protocol: statistical analysis plan for a prospective, multicenter, double-blind, adaptive sample size, randomized, placebo-controlled, clinical trial. Trials.

[2] Spoelstra-de man AME, Elbers PWG, Oudemans-van straaten HM (2018). Making sense of early high-dose intravenous vitamin C in ischemia/reperfusion injury. Crit Care.

[3] Hill A, Clasen KC, Wendt S, Majoros ÁG, Stoppe C, Adhikari NKJ, Heyland DK, Benstoem C. Effects of vitamin C on organ function in cardiac surgery patients: a systematic review and meta-analysis. Nutrients. 2019;11:2103. 10.3390/nu11092103 PMC6769534 31487905

[4] Rhodes A, Evans LE, Alhazzani W (2017). Surviving Sepsis Campaign: International Guidelines for Management of Sepsis and Septic Shock: 2016. Intensive Care Med.

[5] Jain M, Chandel NS (2013). Rethinking antioxidants in the intensive care unit. Am J Respir Crit Care Med.

